# Buruli Ulcer Distribution in Benin

**DOI:** 10.3201/eid1103.040597

**Published:** 2005-03

**Authors:** Roch Christian Johnson, Michel Makoutodé, Ghislain Emmanuel Sopoh, Pierre Elsen, Jules Gbovi, Lise Hélène Pouteau, Wayne M. Meyers, Michel Boko, Françoise Portaels

**Affiliations:** * Programme National de Lutte contre I'Ulcère e Buruli, Cotonou, Benin;; † Institut Régional de Santé Publique (IRSP), Ouidah, Benin;; ‡ Institute of Tropical Medicine, Antwerp, Belgium;; § Médecins sans Frontières-Luxembourg, Cotonou, Benin;; ¶ Armed Forces Institute of Pathology, Washington, DC, USA;; # Université d'Abomey, Calavi, Benin

**Keywords:** Mycobacterium ulcerans, Buruli ulcer, letter

**To the Editor:** *Mycobacterium ulcerans* disease, commonly called Buruli ulcer, is an emerging infectious disease in West Africa (1,2). Several forms of Buruli ulcer exist; large, chronic ulcerations or indurated plaques of the skin are the most frequent manifestations of the disease (1), and bone is sometimes involved (3). Little is known about the focal epidemiology of Buruli ulcer; incidence, prevalence, and other data are usually reported at the national or district level (4). These data convey the importance of the disease but do not show the wide variations that occur at the village level within a given district. In 2002, we investigated the disease in an arrondissement (Gnizounmé) in an area in which Buruli ulcer is endemic, the commune of Lalo in Benin. Prevalence rates of Buruli ulcer varied from 0.58 to 32.62 per 1,000 inhabitants of villages in the same arrondissement. For Gnizounmè Arrondissement, the overall prevalence was 10.70 per 1,000 inhabitants. These results confirmed that distribution of Buruli ulcer must be determined at geopolitical divisions lower than district or national levels, as is frequently assumed to be the case.

An inverse relationship exists between the prevalence of Buruli ulcer and distance from the Couffo River, which drains the arrondissement of Gnizounmè. A comparison of the relevant data for Assogbahoué and Tandji villages shows that the number of patients per 1,000 inhabitants increases gradually from 0.58 to 32.62 as the distance from the river decreases from 10 to 1 km.

Recently, aquatic insects have been considered potential vectors of *M. ulcerans* (5,6). These aquatic insects can fly many kilometers from their source (7). This finding may partially explain how patients who live farther distances from their source of water become infected, but not as often as those who live closer. Some water bugs obtained from water collection points along the Couffo River in the village of Tandji were found to be positive for *M. ulcerans* by using PCR with specific insertion sequence *2404* as a target (8).

If we consider domestic water sources in the arrondissement of Gnizounmè, only Tandji (32.62 Buruli ulcer patients per 1,000 inhabitants) used water directly from the Couffo River. Other villages employed protected water sources for domestic purposes (boreholes, cisterns, or piped water from artesian wells). These results are similar to Barker's findings in Uganda, which showed that families who used unprotected sources of water for domestic purposes had higher prevalence rates of Buruli ulcer than those who used boreholes (9). Consequently, besides the possible influence of distance from the river on disease prevalence through potential vectors, such as insects or other factors, we hypothesize that the use of river water for domestic purposes may also play a role in the elevated prevalence of the disease in Tandji village. If this hypothesis is confirmed, preventive public health programs based on strategies that provide protected water supply systems to villages must be developed to reduce the frequency of the disease.

Determining the complex relationship between distance from the Couffo River and the numbers of cases and level of protection of water supply is difficult. Our findings argue for the need to perform additional epidemiologic studies to understand more completely the key factors that determine the distribution of the disease in the entire commune of Lalo.
